# Effects of Magnesium-Doped Hydroxyapatite Nanoparticles on Bioink Formulation for Bone Tissue Engineering

**DOI:** 10.1021/acsabm.4c01418

**Published:** 2025-01-08

**Authors:** Margherita Montanari, Jannika T. Korkeamäki, Elisabetta Campodoni, Samih Mohamed-Ahmed, Kamal Mustafa, Monica Sandri, Ahmad Rashad

**Affiliations:** † Institute of Science, Technology and Sustainability for Ceramics (ISSMC)—National Research Council (CNR), 48018 Faenza, Ravenna, Italy; ‡ Center of Translational Oral Research (TOR), Department of Clinical Dentistry, University of Bergen, 5009 Bergen, Norway; § Bioengineering Graduate Program, Aerospace and Mechanical Engineering, University of Notre Dame, Notre Dame, Indiana 46556, United States

**Keywords:** 3D bioprinting, GelMA, hydroxyapatite morphology, printability, bone marrow stromal cells, osteogenic differentiation

## Abstract

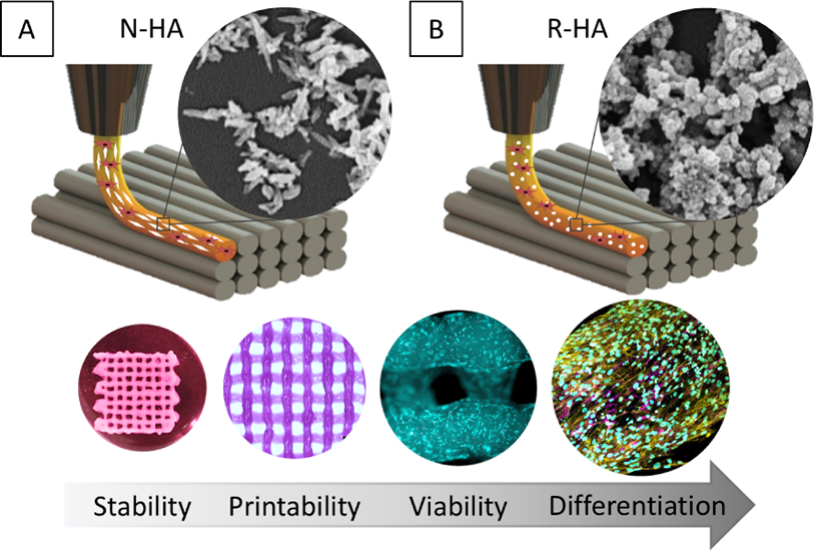

Bioprinting of nanohydroxyapatite (nHA)-based bioinks has attracted considerable interest in bone tissue engineering. However, the role and relevance of the physicochemical properties of nHA incorporated in a bioink, particularly in terms of its printability and the biological behavior of bioprinted cells, remain largely unexplored. In this study, two bioinspired nHAs with different chemical compositions, crystallinity, and morphologies were synthesized and characterized: a more crystalline, needle-like Mg^2+^-doped nHA (N-HA) and a more amorphous, rounded Mg^2+^- and CO_3_^2–^-doped nHA (R-HA). To investigate the effects of the different compositions and morphologies of these nanoparticles on the bioprinting of human bone marrow stromal cells (hBMSCs), gelatin and gelatin methacryloyl (GelMA) were selected as the bioink backbone. The addition of 1% (w/w) of these bioceramic nanoparticles significantly improved the printability of GelMA in terms of extrudability, buildability, and filament spreading. The biological potential of the bioinks was evaluated by examining the hBMSC viability, metabolic activity, and osteogenic differentiation over 21 days. Both nHAs showed high cell viability, with N-HA showing a significant increase in metabolic activity under nonosteogenic conditions and R-HA showing a notable increase with osteogenic stimulation. These results suggest that the two nHAs interact differently with their environment, highlighting the importance of both the chemistry and morphology in bioink performance. In addition, osteogenic differentiation further highlighted how the physicochemical properties of nHAs influence osteogenic markers at both the RNA and protein levels. Clearly, tailoring the physicochemical properties of hydroxyapatite nanoparticles is critical to developing more biomimetic bioinks with great potential for advancing bone bioprinting applications.

## 1 Introduction

Bone tissue is composed of cells and an extracellular matrix (ECM), which can be simplified into two primary components: a mineral phase, composed of nanohydroxyapatite (nHA), and an organic phase, composed mainly of collagen.^1^ Although widely used in tissue engineering research, synthetic nHA (Ca_10_(PO_4_)_6_(OH)_2_) is not a completely accurate representation of the mineral phase of bone. In natural bone tissue, nHA is not stoichiometric and instead can be substantially substituted by the presence of other ions (e.g., HPO_4_^2–^, CO_3_^2–^, Cl^–^, F^–^, Na^+^, Zn^2+^, Sr^2+^, Si^2+^, Mg^2+^).^2,3^

Among the various ions, a high concentration of Mg^2+^ is known to be crucial for bone formation, although its levels significantly decrease in mature bone. Moreover, magnesium deficiency has adverse effects on natural bone, ranging from decreased osteoblastic and osteoclastic activities to fragile bone and even the halting of bone growth.^4^ In addition, Mg^2+^ has been found to accelerate HA nucleation kinetics and inhibit its crystallization process, improving bone resorbability and consequent fast remodeling.^5^ Moreover, in bone tissue engineering, magnesium-based nanoparticles have been reported to stimulate the osteogenic differentiation of mesenchymal stem cells within a 3D scaffold environment.^6^ Thus, the use of ion-doped nHAs in general, and magnesium-doped nHAs in particular, in bone tissue engineering may provide greater benefits and be more biomimetic compared with stoichiometric nHAs.

Extrusion-based 3D bioprinting is an extremely promising method for fabricating living bone-like implants in tissue engineering. The technology is versatile for a wide range of material properties and has fine macro- and micro-geometry control. Additionally, it is more efficient for producing large, personalized implants within a reasonable time frame compared to other printing methods. The main challenge in extrusion-based bioprinting is the development of an optimal ink, referred to as “biomaterial ink” when only materials are used, or “bioink” when living cells are incorporated into the composition.^7^ Important considerations in the development of bioinks are viscosity, printability, postprinting structural stability, and the cytocompatibility of the cross-linking methods. Therefore, when discussing bioinks intended for large constructs, shear-thinning bioinks with self-standing properties and high shape fidelity postprinting are preferred. Moreover, a bioink should protect the embedded cells from excessive shear stresses during the printing and provide the appropriate cues to support cell proliferation, migration, and tissue maturation inside the bioink after bioprinting.^7 −10^

Gelatin methacryloyl (GelMA), a collagen derivative biomaterial, is a commonly studied hydrogel for bioprinting applications, including cartilage, skin, and bone.^11^ As a bioink component, GelMA is considered advantageous due to its easy and fast photo-cross-linking mechanism when combined with a photoinitiator.^12,13^ However, GelMA faces instability and printability issues in bioprinting, which can be overcome with either a high concentration or an additive. To avoid the drawbacks of using high concentrations of GelMA, which can interfere with cell activity due to increased hydrogel stiffness, a sacrificial gelatin can be added to the bioink to improve printing with low concentrations of GelMA.^14^ This is due to gelatin’s reversible thermoresponsive cross-linking properties. Regarding bone tissue applications, GelMA-based hydrogels modified with various versions of calcium phosphate ceramics are widely investigated, ranging from coatings on implants^15^ to 3D bioprinting of biomaterial inks^16^ and bioinks.^17−19^ Although stoichiometric nHA composites are available as bioinks, in-depth studies of their biological responses are still lacking.^20^ The use of ion-doped calcium phosphate ceramics has been reported in only a few studies. Leu Alexa et al. described the potential of Mg^2+^ -doped hydroxyapatite in a GelMA biomaterial ink bioprinted scaffold,^21^ whereas Kim et al. bioprinted two concentrations of synthesized amorphous calcium magnesium phosphate in a GelMA bioink.^22^ However, the existing literature on bioink development is limited to the role of differently doped nHAs, presenting different crystallinity and morphology. Consequently, these nanoparticle properties may interact differently with the polymeric matrix of the inks, affect the 3D bioprinting process in terms of rheology, and influence the biological behavior of the bioprinted cells by altering viscosity, viscoelastic properties, and ion release profiles.

In this study, two biomimetic, differently substituted nanohydroxyapatites were synthesized and evaluated as potential bioceramic components for a GelMA/gelatin bioink. A needle-like nHA (N-HA) doped with Mg^2+^ ions and round-shaped nHA (R-HA) doped with Mg^2+^ and CO_3_^2–^ ions^23,24^ were prepared and characterized. Next, their impact on the printing of GelMA-based inks as well as on the bioprinting of human bone marrow stem cells (hBMSCs) was evaluated. More specifically, the synthesized nHAs were characterized by chemical composition, crystallinity, carbonation, and morphology. In addition, their surface-to-volume ratio was mathematically modeled. The constituted biomaterial inks were investigated in terms of their rheology and printability. The biological response of the printed cells was studied in terms of viability, metabolic activity, and osteogenic differentiation.

## 2 Materials and Methods

### 2.1 Synthesis and Characterization of Biomimetic Nonstoichiometric nHAs

Two different nanohydroxyapatites (nHAs), with different chemical and morphological structures, were synthesized according to the protocol reported by Landi et al.^23^ Briefly, N-HA was synthesized through a neutralization method based on the controlled dripping of 49.6 mL of 1.2 M H_3_PO_4_ acid solution (85% pure, Sigma-Aldrich, St. Louis, MO, USA) into 82.7 mL of 1.2 M Ca(OH)_2_ (95% pure, Sigma-Aldrich) aqueous basal suspension containing 8.48 g of MgCl_2_·6H_2_O (0.42% doping rate), maintained at 40 °C under magnetic stirring. The synthesis of R-HA was identical to that of N-HA, with the addition of simultaneous controlled dripping of 46.0 mL of 0.8 M solution of NaHCO_3_ (Sigma-Aldrich) into Ca(OH)_2_ suspension. The reaction was performed at a lower temperature (25 °C) than N-HA. The differences in synthesis temperature and ion content were set to obtain an effect on the degree of crystallinity and morphology of the nanoparticles. At different ends of the spectrum, a higher temperature leads to more crystalline nanoparticles, whereas using the combination of Mg^2+^ and CO_3_^2–^ doping ions aids in synthesizing more amorphous and resorbable nanoparticles. The precipitation products were aged for 24 h at 25 °C, washed through centrifugation for three times, lyophilized, sieved at 150 μm, then micronized to 3 μm.

Inductively coupled plasma-optical emission spectrometry (ICP-OES) analysis (ICP-OES 5100, vertical dual-view apparatus, Agilent Technologies, Santa Clara, CA, USA) was performed to determine the nHA’s chemical composition and stoichiometry deviations.

X-ray diffraction (XRD) analysis was employed to evaluate the nHA’s crystallographic identity and crystallinity, and the XRD patterns were recorded with a D8 Advance Diffractometer (Bruker, Karlsruhe, Germany) equipped with a Lynx-eye position-sensitive detector, using the CuKα radiation (λ = 1.54178 Å) generated at 40 kV and 40 mA. XRD patterns were acquired in the 10–80° (2θ) range with a step size of 0.02° and a scanning speed of 0.5 s.

Attenuated Total Reflection −Fourier Transform Infrared Spectroscopy (Thermo Nicolet-Avatar 320 iD7 ATR FT-IR, Thermo Fisher Scientific Inc., Waltham, MA, USA) was used to confirm the actual carbonation of R-HA. All the spectra are the average of 64 spectra, collected at room temperature in the wavelength range of 400–4000 cm^–1^ at a resolution of 4 cm^–1^. Three measurements per group were taken (*n* = 3). Spectral data analysis, baseline correction, normalization, and band area were recorded using PerkinElmer Applications Spectrum software.

Powders’ morphology was assessed by electron scanning microscopy (SEM, Carl Zeiss Sigma NTS Gmbh, Öberkochen, Germany) after Au coating (QT150T, Quorum Technologies Ltd., UK).

Thermogravimetric analysis (TGA, STA 449/C Jupiter, Netzsch, Germany) was used to calculate the residual mass of the nHAs, and in particular, to indirectly calculate the carbonation of R-HA.

Simplified mathematical modeling of N-HA and R-HA particles was executed through geometry. To calculate the surface/volume (*S/V*) ratio for each morphology, the following equations were used:
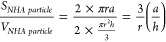
1where *r* is the radius, *h* is the height, and *a* is the apothem of a double cone-shaped N-HA, and
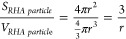
2where *r* is the radius of a spherical R-HA.

### 2.2 Biomaterial Ink Formulation and Printability

Gelatin (Gel) from porcine skin (strength 300, Type A, Sigma-Aldrich) was dissolved in Dulbecco’s Modified Eagle’s Medium (DMEM; Life Technologies, Gibco, Carlsbad, CA, USA) with 1% penicillin-streptomycin (P/S; 10 000 U/mL, Thermo Fisher Scientific, Gibco) at 80 °C in a water bath under magnetic stirring for 30 min, covered to avoid evaporation. The temperature was lowered to 45 °C, and GelMA (gelatin strength 90–110 g bloom, degree of substitution 60%, Sigma-Aldrich) was added to the mixture. Next, the temperature was lowered to 37 °C, and 1 mL of DMEM was used to homogeneously suspend the nHAs before their addition to the polymer mixture. The final composition of the biomaterial ink resulted in 5% w/w_tot_ Gel, 5% w/w_tot_ GelMA, and 0 or 1% w/w_tot_ nHA. The biomaterial inks were finally thermally conditioned as reported in Table 1 before printing at 27 °C.

**Table 1:** Biomaterial Ink Formulation and Conditioning Steps

Ink preparation			Ink thermal conditioning before printing		
Temperature (°C)	Time	Additions	Temperature (°C)	Time (min)	Additions
80	30 min	Gel	37	30	(for bioink constitution)
45	30 min	GelMA	4	18	
37	30 min	nHA (N-HA or R-HA)	27	30	
4	overnight				

The printability of the biomaterial inks was assessed with several experiments: filament drop, buildability, pore shape, and filament spreading, as described previously.^10,25,26^ Samples were designed using Perfactory RP parametric (EnvisionTEC, Gladbeck, Germany) and printed with a 3D-Bioplotter (EnvisionTEC). The tests were executed with an Ø_ID_ = 0.51 mm metal needle (Nordson EFD, Westlake, OH, USA). The printing parameters for the prepared biomaterial inks were as follows: 1 bar and 11 mm/s for GelMA/Gel and 0.5 bar and 11.3 mm/s for the nanohydroxyapatite inks.

For the filament drop test, the filament extrusion profile of each biomaterial ink, extruded at the same pressure, was qualitatively evaluated. The buildability of each ink was assessed by printing two different 3D designs: a hollow cylinder and a hollow cone, both 10 mm in diameter and 20 mm high. For filament spreading estimation, 30 × 30 mm square samples, with 2 layers, 0/90° angle, and a 1.4 mm strand distance, were printed, and high-resolution images of the printed structures were taken. To semi-quantify the biomaterial ink printability through pore shape, as introduced by Ouyang et al.,^27^ Fiji-ImageJ was used to extract the circularity (*C*) data from the high-resolution images (*N* = 1, n_data points_ = 147). The printability factor (Pr) is an estimation of the squareness of a shape and can be quantified with the following equation:
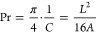
3where *C* is the circularity of a pore, calculated with the perimeter *L* and area *A*. Here, an ideal ink would produce perfectly square-shaped pores, giving a Pr value of 1. However, should an ink obtain too liquid-like properties or present inefficient pregelation, the printed layers would fuse into one another, spread, and leave more circular pores, Pr < 1. On the other hand, if an ink is too gelated or the pore shapes are otherwise irregularly shaped irregularly, Pr > 1. To add more value to the pore shape data, the pore area and distribution were also analyzed in relation to the theoretical model. Results are presented as representative images from multiple printed models.

### 2.3 Biomaterial Ink Rheology

The rheological properties of the biomaterial inks were investigated with a C-VOR 120 rotational rheometer C-VOR 120 (Bohlin Instruments, UK). The flow sweep test was performed to determine the viscosity of the biomaterial inks. The measurements were executed with a plate/plate PP20 (Ø = 20 mm) geometry under increasing shear rate (10–10000 s^–1^) at a constant temperature (27 °C). After sample loading, the samples were left to rest for 3 min to reduce any possible influence on the measurement due to solution handling. The measurements were performed by using a solvent trap to avoid water evaporation during the test.

### 2.4 Cell Isolation, Characterization, and Culture

After patient consent and approval from the Regional Committee for Medical and Health Research Ethics (REK) in Norway (2013/1248/REK, 2024/704736/REK), human bone-marrow-derived mesenchymal stromal cells (hBMSCs) were isolated from young donors (9–14 years old) undergoing corrective surgery at the Department of Plastic Surgery, Haukeland University Hospital, Bergen, Norway. Full characterization of the cells was published in our previous report.^28^ The stemness of the cells was confirmed following recommendations from the International Society for Cellular Therapy (ISCT).^29^ The isolated cells demonstrated strong expression (≥99%) of CD73, CD90, and CD105 surface markers and negative expression (≤2%) of hematopoietic markers CD34, CD45, and HLA-DR, thus verifying the mesenchymal origin of the stem cells.

Cells were cultured and expanded in CellStack (Nunc, Thermo Fisher Scientific) to reach the desired number of cells, under humidified 5% CO_2_ at 37 °C in basal media (BM) composed of DMEM with 10% fetal bovine serum (FBS; HyClone, GE Healthcare, Utah, U.S.A.),1% penicillin–streptomycin (P/S; 10 000 U/mL, Thermo Fisher Scientific, Gibco) (basal medium; BM) and supplemented with 5 ng/mL human FGF-2 (Miltenyi Biotec, Bergisch Gladbach, Germany).

### 2.5 Bioprinting and Cross-Linking

To prepare the biomaterial inks for cross-linking, sterile-filtered lithium phenyl-2,4,6-trimethylbenzoylphosphinate (LAP, >98%, TCI, Tokyo, Japan) was added (stock solution 10 mg/mL), and the whole solution was mixed for 1 h at 37 °C in the dark, covered to avoid evaporation, reaching the final LAP concentration of 0.1% w/*w*_tot_.

In order to constitute a bioink, cells were detached from the flasks, centrifuged, resuspended in BM, and finally mixed with the ink solution using a Luer-lock-connected double syringe system. The final cell concentration in the bioink was 5 × 10^6^ /mL. After constitution, the bioinks were thermally conditioned as reported in Table 1.

The samples (10 × 10 mm, 0/90° angle, 1.2 mm strand distance) were bioprinted with the 3D-Bioplotter (EnvisionTEC) directly into 6-well plates placed onto a 4 °C printing platform and protected from light. All structures were 4 layers in height and were printed with a Ø_ID_ = 0.51 mm metal needle using an average pressure of 1.3 bar at an average speed rate of 13 mm/s.

Immediately after printing, structures were cross-linked with a dental curing lamp (Bluephase G2, Bluephase LED curing light, high energy mode) for 30 s with a perpendicular exposure at a 0.5 cm distance. Scaffolds were then moved into 24-well plates and cultured in BM or osteogenic medium (OM; BM supplemented with l-ascorbic acid (173 μM), dexamethasone (10 nM), and β-glycerophosphate (10 mM) (Sigma-Aldrich, St. Louis, MO, USA)) for 21 days. Normocin (InvivoGen, Toulouse, France) was added under all medium conditions.

### 2.6 Evaluating the Biological Effects of nHAs on 3D Bioprinted Structures

The bioprinted cells were evaluated for their viability, metabolic activity, and osteogenic differentiation over a period of 21 days.

Cell viability was assessed by the live/dead (L/D, Live/Dead Viability/Cytotoxicity Kit for mammalian cells, Molecular Probes, Invitrogen) staining test, and the images were taken with a fluorescence microscope (Nikon Eclipse Ti, Tokyo, Japan) as previously described.^30^

For cell proliferation (*n* = 5), metabolic activity was used as an indirect indicator using Cell Counting Kit-8 (CCK-8, Dojindo Laboratories, Kumamoto, Japan) according to the manufacturer’s instructions. The samples were supplied with 200 μL of the working solution and incubated for 2 h at 37 °C in 5% CO_2_. The absorbance was read at 450 nm with a microplate reader (Varioskan LUX, VLBL00D0, Thermo Fisher Scientific). Results were presented in absorbance units and relative to each group’s day 1 time point. Statistical analysis was performed on absorbance values.

Osteogenic gene expression in the bioprinted samples was evaluated at D7 and D21. The following genes were chosen as indicators of osteogenic commitment: runt-related transcription factor 2 (*Runx2*, Hs01047973_m1), alkaline phosphatase (*ALP*, Hs01029144_m1), and osteocalcin (*Ocn*; BGLAP, Hs01587814_g1) (Thermo Fisher Scientific, Waltham, MA, USA). Glyceraldehyde-3-phosphate dehydrogenase (GAPDH, Hs02758991_g1, Thermo Fisher Scientific, Waltham, MA, USA) was utilized as an endogenous control. At time points, samples (*n* = 5) were collected, washed with PBS, and frozen to −80 °C prior to RNA extraction. On the day of processing, the samples were thawed and dissolved with 1.5 mg/mL Collagenase I (195 u/mg, Worthington Biochemical Corporation, Lakewood, NJ, USA) in nuclease-free water at 37 °C for 1 h. After the structures were dissolved, the samples were processed with TRIzol Reagent Invitrogen (Thermo Fisher Scientific, Waltham, MA, USA) as per the manufacturer’s instructions. Shortly after the addition of TRIzol, samples were sonicated to lyse and homogenize the samples. Next, samples were incubated at 25 °C to allow for complete dissociation of the nucleoproteins. Afterward, chloroform was added, and the samples were vortexed to break down nuclei and nucleic acid complex structures. Finally, samples were incubated for 3 min and centrifuged, and the upper aqueous phase formed in each sample Eppendorf containing the RNA was carefully collected. To precipitate the RNA, isopropanol was added, and the samples were incubated for 10 min and then centrifuged. RNA resulted in a white pellet at the bottom of the Eppendorf. The supernatant was discarded, and the RNA pellet was washed three times by resuspending it in 75% ethanol and centrifuging for 10 min at 12000 rpm. The pellet was finally dissolved in 40 μL of RNase-free water. RNA quality and concentration were measured with a spectrophotometer (Nanodrop ND-1000, Nanodrop Technologies, Wilmington, DE, USA). Next, complementary DNA was synthesized with a High-Capacity cDNA Reverse Transcription Kit (Applied Biosystems, Thermo Fisher Scientific, Waltham, MA, USA). Lastly, quantitative real-time polymerase chain reaction (qRT-PCR) was performed with the StepOne RT-PCR System (Applied Biosystems, Thermo Fisher Scientific, Waltham, MA, USA) and TaqMan Fast Universal PCR Master Mix (Applied Biosystems, Thermo Fisher Scientific, Waltham, MA, USA). All measurements were executed with technical replicates. Relative quantification of gene expression was performed using 2^(−ΔΔCt)^ method and expression levels are presented as relative to the control sample cultures.^31^ Statistical analysis was performed on the mean ΔCt values.

Immunofluorescence analysis, modified from Honkamäki et al.^32^ and Mykuliak and Yrjänäinen et al.,^33^ was used to detect the protein expression of *Ocn* at D21. Bioprinted scaffolds were fixed with 4% paraformaldehyde (pFA, Sigma-Aldrich) for 1 h at room temperature, washed twice with DPBS for 10 min, permeabilized with 0.3% Triton X-100 in DPBS for 10 min, and blocked with 0.1% Triton X-100, 1% BSA, and 10% normal goat serum (NGS) in DPBS overnight. After a series of washes with 1% NGS, 0.1% Triton X-100 and 1% BSA in DPBS, samples were incubated under shaking with mouse monoclonal osteocalcin (*Ocn*; Novus Biologicals, Littleton, CO, USA; dilution 1:200 in 1% NGS and 1% BSA in DPBS) for 2 days at 4 °C. Three series of washes with 0.1% Triton X-100 and 1% BSA in DPBS were executed, and then samples were left in the washing solution overnight at 4 °C under shaking. Goat antimouse Alexa Fluor 647 IgG as a secondary antibody (Life Technologies, Carlsbad, CA, USA, dilution 1:400 in 1% BSA in DPBS) was applied together with the actin cytoskeleton staining using phalloidin tetramethylrhodamine-B isothiocyanate (Sigma-Aldrich, dilution 1:500 in 1% BSA in DPBS). After washing with DPBS, the nuclei were stained with 4′,6-diamidino-2-phenylindole (DAPI) (Sigma-Aldrich, dilution 1:2000 in DPBS) for 15 min. Images were taken using a confocal microscope (Leica TCS SP8 STED 3X, Leica Microsystems, Germany) and processed with Fiji ImageJ software.^34^

### 2.7 Statistical Analysis

Pore area and metabolic activity data are presented as mean ± SD. For metabolic activity and gene expression, the statistical analysis was performed using IBM SPSS Statistics (Version 28.0.1.1). One-way ANOVA with Tukey’s post hoc was used for significance testing when comparing the three groups to one another. A paired Student’s *t*-test was used for significance testing between BM and OM cultures. The differences were considered statistically significant for *p* < 0.05.

### 2.8 Data Availability Statement

The datasets generated and analyzed for this study are available from the corresponding author upon reasonable request.

## 3 Results and Discussion

### 3.1 Synthesis and Characterization of Biomimetic Nonstoichiometric nHAs

The two chosen physicochemically and morphologically different nanohydroxyapatites (nHAs) were prepared and characterized. The effective ion substitution was confirmed by the ICP analysis presented in Table 2. The typical Ca/P ratio of stoichiometric HA of 1.67 was decreased to 1.51 for the N-HA as a result of successful Mg^2+^ doping, and 1.55 for R-HA. In addition, the (Ca+Mg)/P ratio was recorded as 1.78 for N-HA and slightly higher at 1.85 for R-HA. This is in agreement with several reports demonstrating the synergistic interaction of Mg^2+^ and CO_3_^2–^ toward the doping during the synthesis.^23,24^ In addition, the difference in the concentration of CO_3_^2–^ was recorded with TGA.

**Table 2:** Chemical Composition and Morphology of the Prepared nHAs, as Evaluated Using ICP, TGA, and SEM

Label	Doping ions	Synthesis T (°C)	Ca/P	(Ca + Mg)/P	CO_3_^2–^ (wt %)	Morphology
N-HA	Mg^2+^	40	1.51 ± 0.01	1.78 ± 0.01	1	Needle
R-HA	Mg^2+^/CO_3_^2–^	25	1.55 ± 0.01	1.85 ± 0.00	2.46	Round

**Figure 1:**
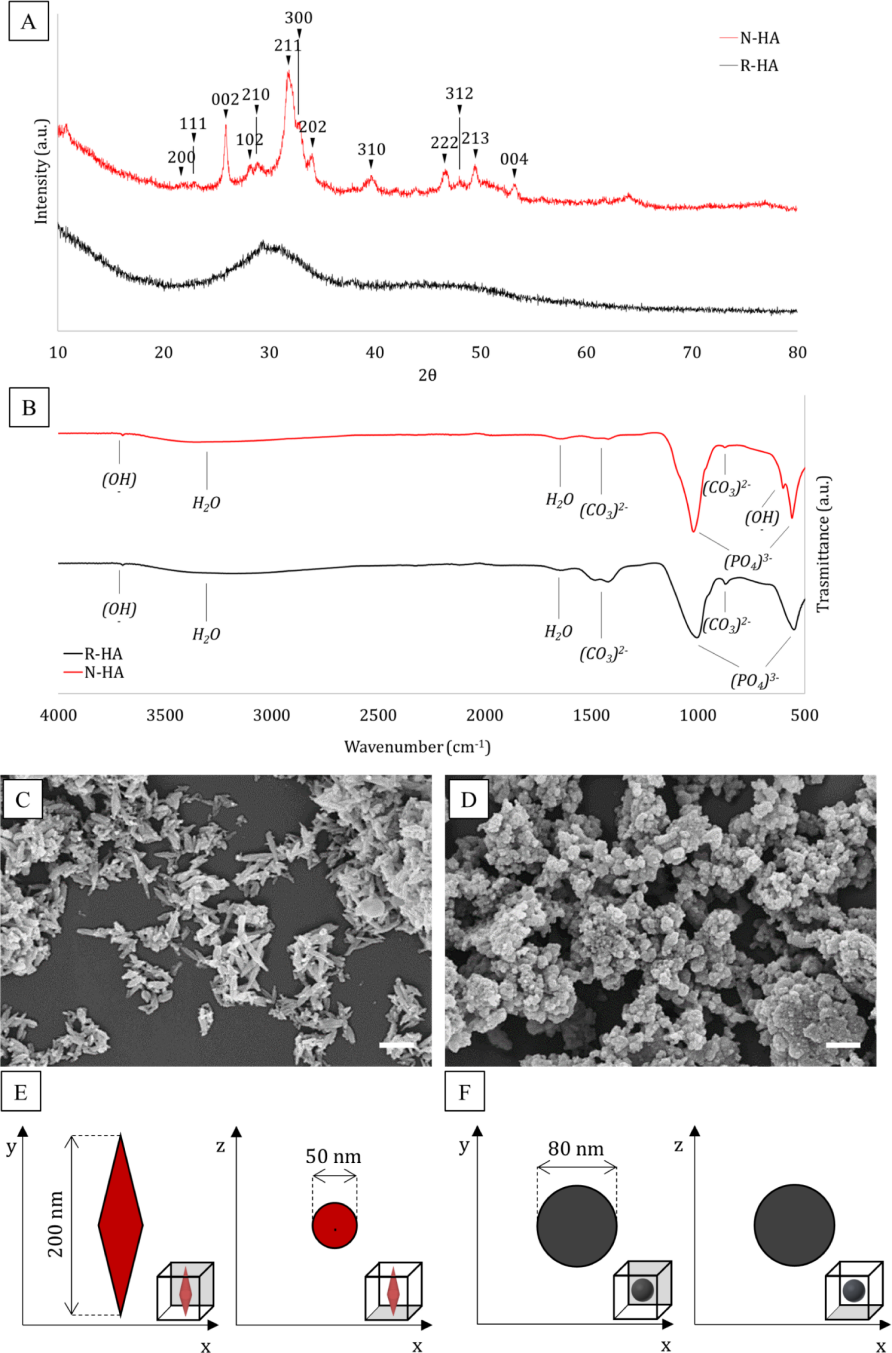
Physicochemical and morphological characterizations of nHAs. (A) XRD spectra of N-HA (red) and R-HA (black); (B) ATR-FTIR spectrum of R-HA (black) and N-HA (red); (C) SEM images of N-HA; and (D) R-HA, scale bar 200 nm; (E) simplified geometrical rendering of N-HA and (F) R-HA.

X-ray diffraction was employed to assess the nHA identity and crystallinity degree. The identity was demonstrated by HA peak recognition for N-HA as shown in Figure 1A. N-HA showed a higher degree of crystallinity compared to the R-HA, which was almost amorphous, as indicated by the poorly resolved spectra.

ATR-FTIR analysis (Figure 1B) revealed distinct phosphate bands in the ranges of 950–1050 cm^–1^ and 550–600 cm^–1^, together with the presence of both the absorbed and the occluded water, indicated by the broad band around 2650–3650 cm^–1^ and the peak at 1660 cm^–1^, respectively. Moreover, the typical signals of β-carbonation, indicating substitution at the phosphate site, were detected, as shown by the presence of CO_3_^2–^ stretching signals around 1420 and 1480 cm^–1^ and the bending peak around 870 cm^–1^. As expected from the synthesis design, R-HA showed significantly higher carbonation compared to N-HA, which showed low carbonation, probably due to the environmental conditions during synthesis.^5,23,35^ It was possible to obtain two different types of nanoparticles by exploiting the synergistic effect resulting from the different synthesis temperature (25°C instead of 40°) combined with the simultaneous introduction of substituent ions (e.g., Mg^2+^ and CO_3_^2–^) into the hydroxyapatite lattice, both of which are responsible for not promoting the crystallization process.

Two key factors substantially influence the solubility of nHAs under physiological conditions: the degree of crystallinity and the doping ions (type and quantity), which help make the synthesized nHA resemble natural bone tissue.^23,36^ In the present work, MgCl_2_, which is also a constituent of synthetic body fluid, was used to synthesize Mg^2+^ and Mg^2+^/CO_3_^2–^ biological-like substituted nHAs. The classical neutralization route involving calcium hydroxide and ortho-phosphoric acid produced a monosubstituted medium crystalline and needle-like hydroxyapatite, N-HA. Instead, a cosubstitution with CO_3_^2–^ ions resulted in almost amorphous and round-shaped R-HA, exploiting the ability of CO_3_^2–^ to promote Mg^2+^ entrance in nHA crystal lattice and to consequently act as a crystallinity and morphology tuner.^23^ Therefore, based on the results, it could be expected that the N-HA, with its higher degree of crystallinity, degrades slower in physiological conditions. In contrast, R-HA could be more resorbable, which is a crucial feature in the development of new materials addressing bone regeneration.^23,37,38^

SEM analysis was performed to assess the morphology and particle dimensions (Figure 1C,D). The higher temperature (40 °C) and single-ion substitution of N-HA resulted in a needle-like morphology (Figure 1C), with particles approximately 200 nm in length and 50 nm in width. On the other hand, the lower temperature (25 °C) and double substitution of R-HA produced a round-shaped morphology, with particles of approximately 80 nm in diameter (Figure 1D). Elaborating from these particle dimensions and shapes obtainable from the morphological analysis, the N-HA particle was reduced to a double cone (Figure 1E) and the R-HA particle to a sphere (Figure 1F) using a simplified geometrical rendering.

The variations in the shape and dimensions of the synthesized nHAs resulted in a difference in the estimated surface area, and therefore contact area: the surface area of N-HA was approximately 80% of that of R-HA. This variation may affect resorption and, more importantly, cellular response. On the other hand, the needle and sphere may have different effects on the biomaterial ink flow and, therefore, viscosity and the bioprinting process.

### 3.2 Rheology and Printability

**Figure 2:**
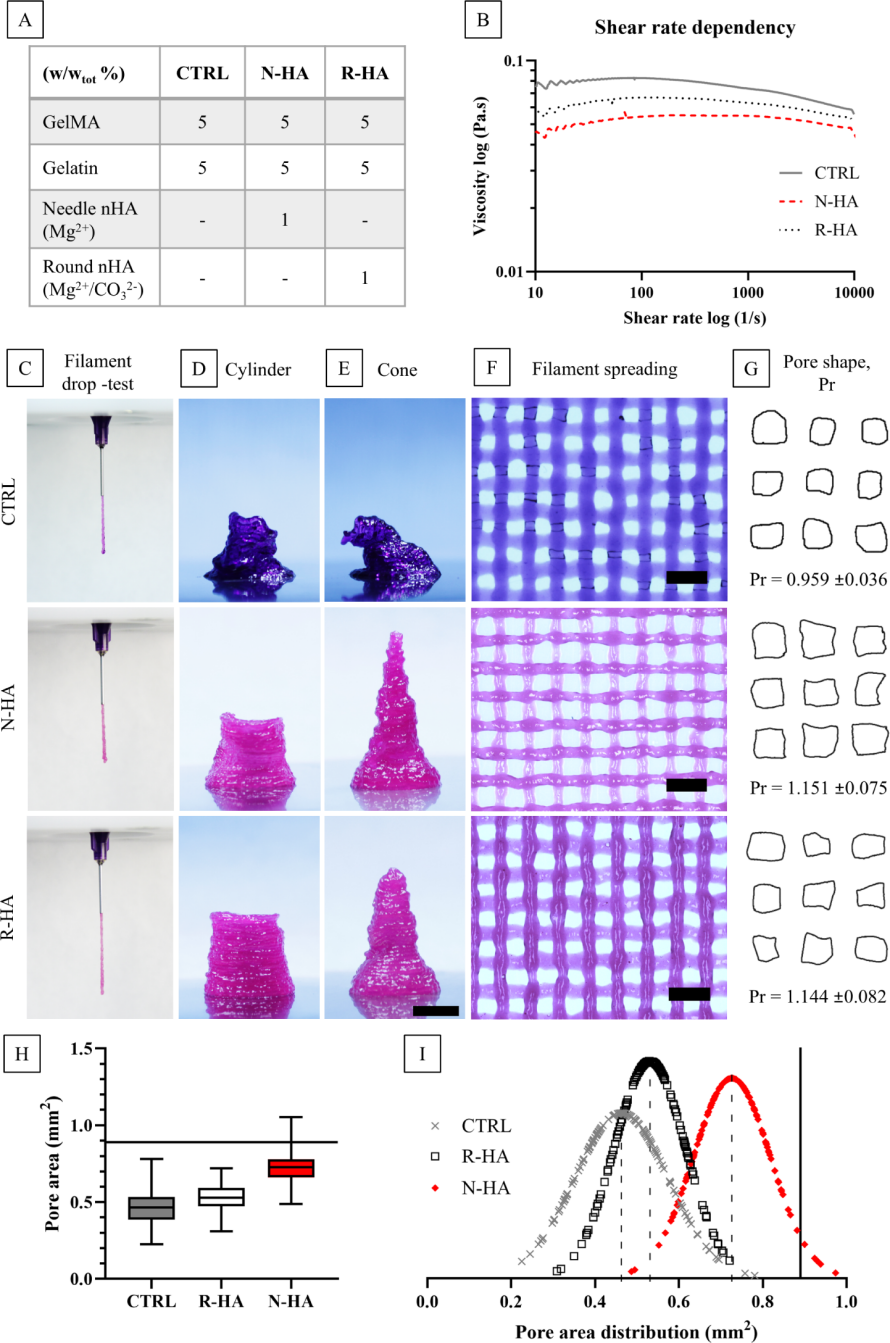
(A) Biomaterial ink compositions. (B) Shear rate dependency of CTRL, N-HA, and R-HA biomaterial inks. (C–I) Printability evaluation of CTRL, N-HA, and R-HA biomaterial inks: (C) filament drop test, buildability for (D) hollow cylinder and (E) cone structures, scale bar 5 mm, (F) filament spreading, scale bar 2 mm, (G) calculated Pr values (*n* = 147) and pore shape illustrations, and (H), and (I) CTRL, N-HA, and R-HA 3D printed scaffold pore size area comparison and distribution. In (H) and (I), the theoretical model is presented as a black line (*n*_data points_ = 147) and means are presented as dashed line.

The composition of all three biomaterial inks is presented in Figure 2A and their shear rate dependency is illustrated in Figure 2B. At the printing temperature (27 °C), all the biomaterial inks demonstrated a relatively low viscosity throughout the shear rate range. However, small differences are found between the groups. Interestingly, the data revealed a slightly higher viscosity for the CTRL than for the nHA compositions. In addition, R-HA had a slightly higher viscosity throughout the shear rate range in comparison to N-HA. Moreover, the incorporation of nHA into a GelMA/Gelatin biomaterial ink is considered to increase the sol–gel transition temperature slightly (unpublished data). It could be speculated that the GelMA and gelatin thermal gelation network (order of magnitude: 10^–10^ m; e.g., the average length of an H-bond of about 0.2 nm) is physically impaired by the nHA particles (order of magnitude: 10^–9^ m; e.g., N-HA 200 nm length). On the other hand, although the biomaterial ink preparation aims for a homogeneous paste, the possible nHA particle size variation and agglomerates in the ink might create a liquid–solid-like environment.^39^ In other words, the ceramic phase in an ink could lead to phase separation under pressure, leading to faster liquid flow than in the solid phase under extrusion.^30,40^

The difference between the two nHAs could be explained through simplified geometrical modeling. When considering the geometrical rendering (Figure 1E,F) and eqs 1 and 2, the *S/V* ratio for N-HA particles is higher than the *S/V* ratio of R-HA particles by 26%. This substantial difference in the *S/V* ratio could create a difference in the physical gelation impairment, leading to a lower viscosity of N-HA.

Printability estimation of the biomaterial inks in terms of extrudability, buildability, and model fidelity is presented in Figure 2C–I. The filament drop test for the developed biomaterial inks showed a steady flow consistent with the nozzle diameter (Figure 2C), with no evidence of swelling, curling, or dripping. This indicates that sufficient pregelation was achieved, and a proper printing temperature was chosen. The filament drop test primarily reflects the initial gelation state of a biomaterial ink, which not only affects the printing quality but can also lead to an uneven distribution of cells in the ink cartridge.^27^

The cylindrical and conical structures printed with the CTRL group (Figure 2D,E), were poorly buildable, collapsing quite early during the printing process. On the contrary, the addition of only 1% w/w_tot_ of nHA was found to be crucial in improving the buildability properties. It could be speculated that the addition of nHA improved the flow of the biomaterial ink by shifting the equilibrium during extrusion from elastic behavior toward plastic, while maintaining a fast temperature-driven setting time, typical of thermosensitive hydrogels.^11^

Based on the semiquantitative filament spreading data (Figure 2F), the quantification of the Pr factor (Figure 2G) revealed that despite its poor buildability, GelMA alone produced the most square-like pores (Pr = 0.959 ± 0.036), while the R-HA and N-HA resulted in values of Pr > 1. According to Ouyang et al.,^27^ all inks were characterized roughly in the “printable region” of 0.9 ≤ Pr ≤ 1.1. However, this model does not consider the accuracy of the pore area, and therefore, in the current study, additional quantifications were made.

The pore area accuracy and distribution were found to be the smallest for the CTRL group, revealing the most spread filaments (Figure 2H,I). However, the addition of 1% w/w_tot_ R-HA improved shape fidelity. Moreover, the addition of N-HA not only improved the pore shape but also improved the pore area accuracy substantially. According to the pore shape (Pr) calculation, the nHA inks demonstrated a more gelated ink (Pr > 1). However, this provided more stability to the filaments and led to more accurate pore sizes. It should be noted that a single printability parameter can only indicate very little about the performance of the biomaterial ink. Therefore, several methods should be applied as this is more useful in biomaterial ink comparison and when moving toward more complex or larger structures.

### 3.3 Structural Stability, Viability, and Metabolic Activity of Bioprinted hBMSCs

Before bioprinting the hBMSCs, the cytotoxic effect of the nHAs was estimated under 2D culture conditions for up to 7 days in BM and OM (SI 1.1). The results indicated low cytotoxicity, with high cell viability observed in both culture conditions (Figure S1A). In addition, the metabolic activity of the nHA-exposed cells increased 2-fold in BM and almost 4-fold in OM (Figure S1B,C).

**Figure 3:**
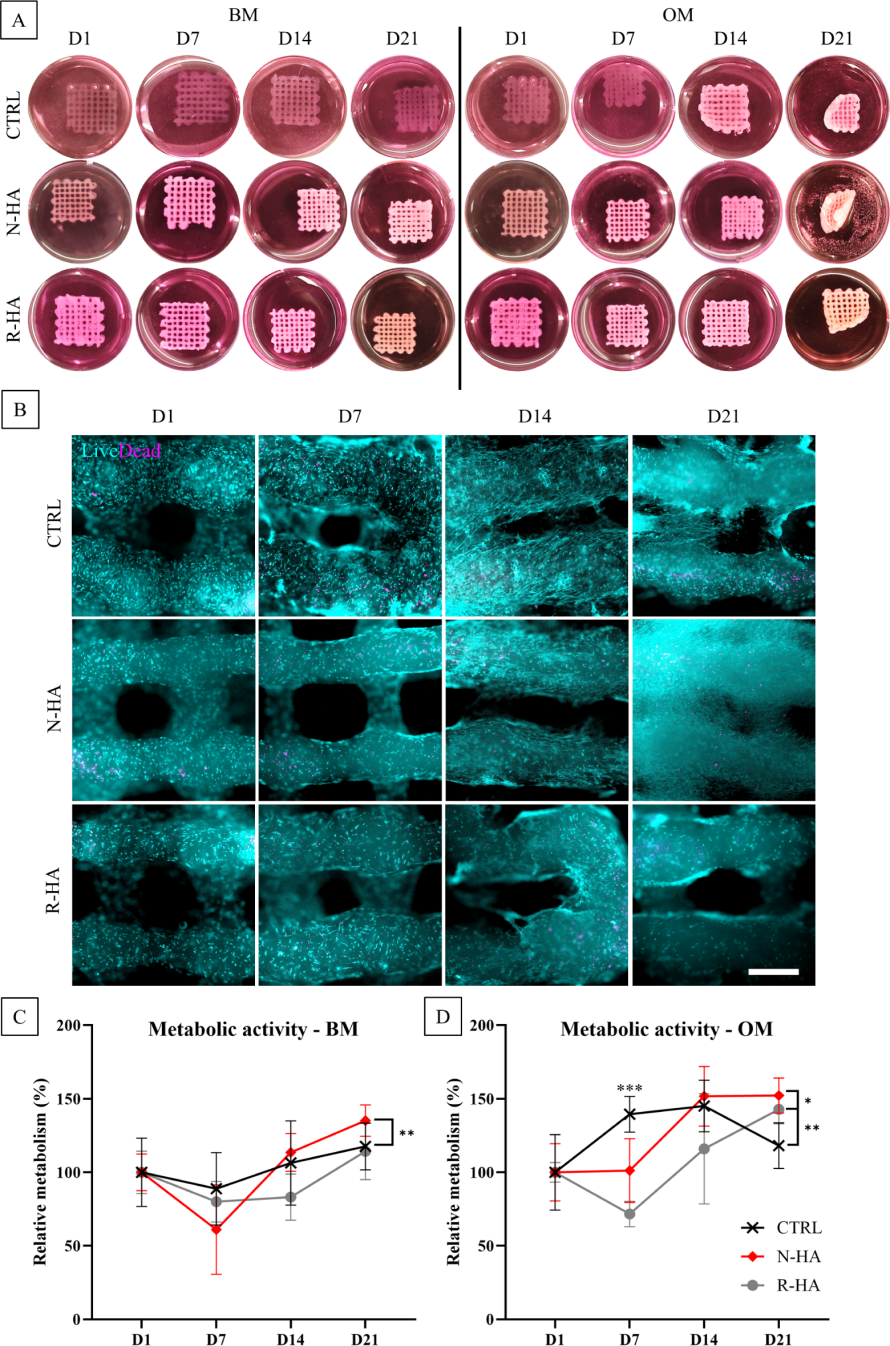
(A) Stability of CTRL, N-HA, and R-HA 3D bioprinted scaffolds at D1, D7, D14, and D21 in BM and OM. Structure dimensions: 10 × 10 mm. (B) Representative focal planes of cell viability. The hBMSCs were cultured in OM: live (cyan), dead (magenta). Scale bar 500 μm. (C) Metabolic activity (*n* = 5) of bioprinted hBMSCs at D1, D7, D14, and D21, cultured in BM and (D) OM. The results are presented relative to each group’s D1. Differences between groups, based on assay absorbance: *p < 0.05, **p < 0.01, ***p < 0.001.

All 3D bioprinted structures, as presented in Figure 3A, demonstrated structural integrity for up to 21 days in both BM and OM. However, two interesting phenomena were observed. The transparent nature of the CTRL samples became increasingly opaque between D7 and D14 in OM. In addition, noticeable scaffold curling was observed at day 21 in all groups cultured in OM. As both changes were specific to OM conditions, this suggests increased cell spreading and significant scaffold colonization. Shape transformation and functional maturation of 3D bioprinted structures over time are considered, in today’s terms, 4D bioprinting.^41^ However, as the shape transformation in the current study occurred spontaneously, the mechanism can be speculated to originate from the combination of material properties and cell activity. Since the structures were cross-linked after printing with a single light projection, it is likely that a cross-linking gradient developed, with the top being stiffer due to more efficient light exposure, while the bottom received less light and therefore less effective cross-linking. In agreement with that, bilayered structural designs with varying material properties, resulting in intentional curling or folding of structures, have been demonstrated in previous studies.^42,43^ However, since the shape transformation only occurred in OM-cultured samples, it is likely that increased cellular activity and the resulting cell traction forces (CTF)^41^ played a role, as previously demonstrated by Kuribayashi-Shigetomi et al.^44^ Although the shape change was independent of the nHA properties, it was more pronounced in the N-HA samples.

High cell viability was demonstrated by the L/D assay throughout the culture period, as shown in Figure 3B. In BM (Figure S2) as well as in OM, at D1, it is possible to see a homogeneous cell distribution within the filaments, indicating a successful bioink constitution. The high viability indicates a suitable combination of the LAP concentration and light exposure. In addition, it can be concluded that shear stress during the bioprinting process did not negatively affect cell viability in the short term. However, slightly more dead cells can be seen in CTRL samples in the BM, showing an increasing trend up to D7. This could possibly relate to the long-term effect of shear stress undergone by the cells during the printing process.^45−47^ From a rheological standpoint, the CTRL samples exhibited higher viscosity compared to that of the nHA-modified inks. Increased viscosity in bioinks has been reported to generate greater shear stress during bioprinting, potentially reducing cell viability and proliferation.^48^ Moreover, because of the transparent nature of CTRL bioinks, the hBMSCs bioprinted in these inks are likely exposed to more light during cross-linking than those in the nHA bioinks. This could be another possible explanation for the reduced cell viability observed in the CTRL samples. More importantly, under both medium conditions, the cells appeared to recover and adopt an elongated morphology. At D14, there were some differences between samples cultured in BM and OM: in OM, the cells appeared to extensively colonize the filaments and bridge the gaps between them. This difference was maintained until D21 and supports the theory that CTF might be involved in the observed shape transformation. The most pronounced hBMSC colonization was found to be in the N-HA group in the OM at D21.

Metabolic activity (MA) results aligned with the viability data (Figure 3C,D). The results were assessed and presented relative to day 1 of each group, as differences in cross-linking between groups may affect reagent permeability.^49^ In general, it appeared that both the composition of the inks and the culture media influenced MA. In the first week, while the MA of cells in the CTRL samples slightly decreased by day 7 in BM, it increased for the cells cultured in OM. Regardless of the culture medium used, cells bioprinted in CTRL bioinks exhibited higher metabolic activity than those in nHA-based bioinks at day 7. For N-HA during the first week, the MA decreased in BM but remained the same in OM, while the R-HA bioink showed a reduction in MA during the first week in both conditions. Early time points recorded plateaus, lags, and decreases in both MA and proliferation have been reported previously with 3D bioprinted cultures during the first 2 weeks.^50,51^ In addition to the lengthy bioprinting procedures, the decrease in MA and cell proliferation can be attributed to the stress experienced by the cells through the bioprinting process. The cells are exposed to shear forces starting from bioink mixing, pressure during bioprinting^48^ and shear stress during extrusion through the needle.^52^ Most importantly, the cells show signs of recovery, and by day 21, the bioink containing N-HA particles exhibited significantly higher MA than CTRL in BM, and the bioink containing R-HA particles exhibited significantly higher MA than other groups in OM (Figures 3C,D and S2B,C).

The differences observed between the N-HA and R-HA groups in the first week may be related to their different physicochemical properties. It can be speculated that the higher MA of N-HA is due to its needle-like morphology and increased S/V ratio, which may affect the hBMSCs under shear stress in the short term. In contrast, the amorphous and more resorbable nature of the R-HA particle, along with its larger surface area, may result in excessive osteogenic stimulation for hBMSCs during the first few days in OM, thereby decreasing their metabolic activity and proliferation rate. However, in the current study, the cells in R-HA-containing bioinks seem to recover from this possible overstimulation over time.

### 3.4 Evaluating the Osteogenic Effects and Potential

**Figure 4:**
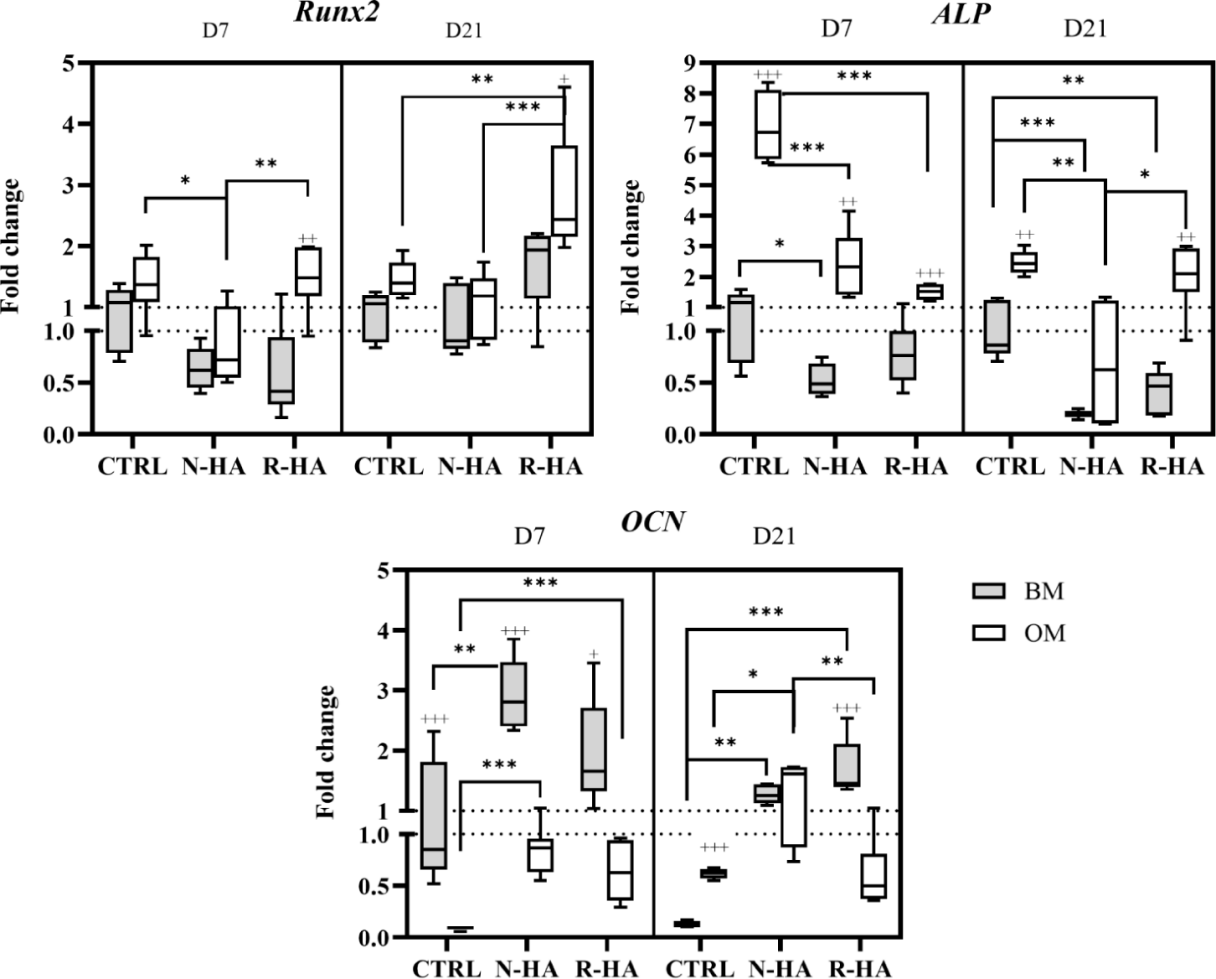
Osteogenic gene expression of the bioprinted CTRL, N-HA, and R-HA samples at days 7 and D21. Gene expression was evaluated for *Runx2*, *ALP*, and *Ocn*. The results are relative to the D7 BM CTRL sample. Differences between groups: *p < 0.05, **p < 0.01, ***p < 0.001; differences between culture conditions: +*p* < 0.05, ++*p* < 0.01, +++*p* < 0.001.

Gene expression of osteogenic markers *Runx2*, *ALP,* and *Ocn* was assessed at D7 and D21 to evaluate the osteoinductive potential of the formulated bioinks (Figure 4). Similar to the MA, it appeared that both the composition of the bioinks and the culture media influenced osteogenic differentiation of the printed cells. On day 7, R-HA samples cultured in OM showed the highest expression of *Runx2*. This was significantly different from the BM culture condition. The expression in N-HA was found to be significantly lower compared with the other groups in OM. *Runx2* expression increased under all groups and conditions from D7 to D21. The greatest increase was observed in R-HA samples cultured in OM, showing a significant difference compared to other groups and the BM culture condition. With regard to *ALP*, a significantly higher level of expression was observed in the CTRL samples on D7 compared to the other groups cultured in OM. In addition, a significant difference was recorded between the culture conditions of each group. A decrease in *ALP* expression was observed in all groups and conditions except R-HA cultured in OM. This decrease is expected because ALP is classically considered an early to mid osteogenic differentiation marker. The peak of *ALP* expression is expected to occur between the proliferation plateau and collagenous ECM formation,^53^ where ALP contributes to the calcification of collagen.^54^ These results are in line with the recorded MA for CTRL samples cultured in OM, where a plateau was recorded from D7 to D14. The same is not demonstrated for the nHA groups and could therefore happen later than D7 but before D21. Moreover, as the R-HA MA plateau is not recorded during the culture period, it could be speculated that late upregulation of *Runx2* and *ALP* expressions together with the MA profile indicate a delayed osteogenic commitment after cell recovery. This is not unusual, as delays in osteogenic expression or mineralization in *in vitro* 3D cultures have been reported^16,17^ in comparison to the golden standard time points used in 2D cultures for osteogenic differentiation.

Surprisingly, high expression levels of *Ocn* were observed on day 7 in all bioinks. As *Ocn* is strongly associated with mineralization at a later stage in osteogenic commitment,^53^ it is speculated that the stress experienced during bioprinting affects the hBMSC functionality already in the short-term. Chawla et al.^55^ demonstrated a significant difference in *Ocn* expression at D5 between bioprinted silk-gelatin-embedded BMSCs and those cultured in 2D. In this study, *Ocn* expression was higher in nHA samples in all conditions compared to CTRL, with significant increases in N-HA cultured in BM and in both nHA samples cultured in OM. This inducing effect of a ceramic filler in GelMA-based bioinks has also been reported in the literature. Alcala-Orozco et al. bioprinted hMSCs encapsulated in strontium-GelMA and recorded a significant increase in *Ocn* in strontium-containing bioprinted structures over the GelMA control on D7.^56^ Similarly, Allen et al. incorporated hydroxyapatite into GelMA and recorded increased *Ocn* expressions over control on D14.^20^ Interestingly, unlike the expression of *Runx2* and *ALP*, the expression of *Ocn* was significantly higher in BM culture conditions compared to the OM samples on D7. The result suggests that the presence of each nHA in the bioink may induce osteogenic differentiation without osteogenic supplements. Supporting this speculation, Dubey et al. demonstrated osteogenic gene expression in amorphous magnesium phosphate/ECM bioprinted structures without the need for osteogenic medium supplements.^57^

When considering the two different nHAs and all three chosen osteogenic gene expression markers, time and culture conditions are important. As R-HA stimulates higher *Runx2* expression, it may indicate early osteogenic commitment but not necessarily complete ECM maturation. In contrast, greater *ALP* expression in the N-HA group may suggest a faster progression of matrix maturation. This hypothesis was supported by the higher *Ocn* expression in the N-HA group. These variations could be attributed to the different physicochemical properties of nHA particles, which may lead to distinct ion release kinetics. The 3D encapsulation of R-HA particles and hBMSCs could create high local concentrations of released ions. This kind of excessive presence of Mg^2+^ was also discussed by Kim et al.^22^ as osteogenic differentiation was inhibited or delayed when higher concentrations of amorphous calcium magnesium phosphate were incorporated into a GelMA bioink.

**Figure 5:**
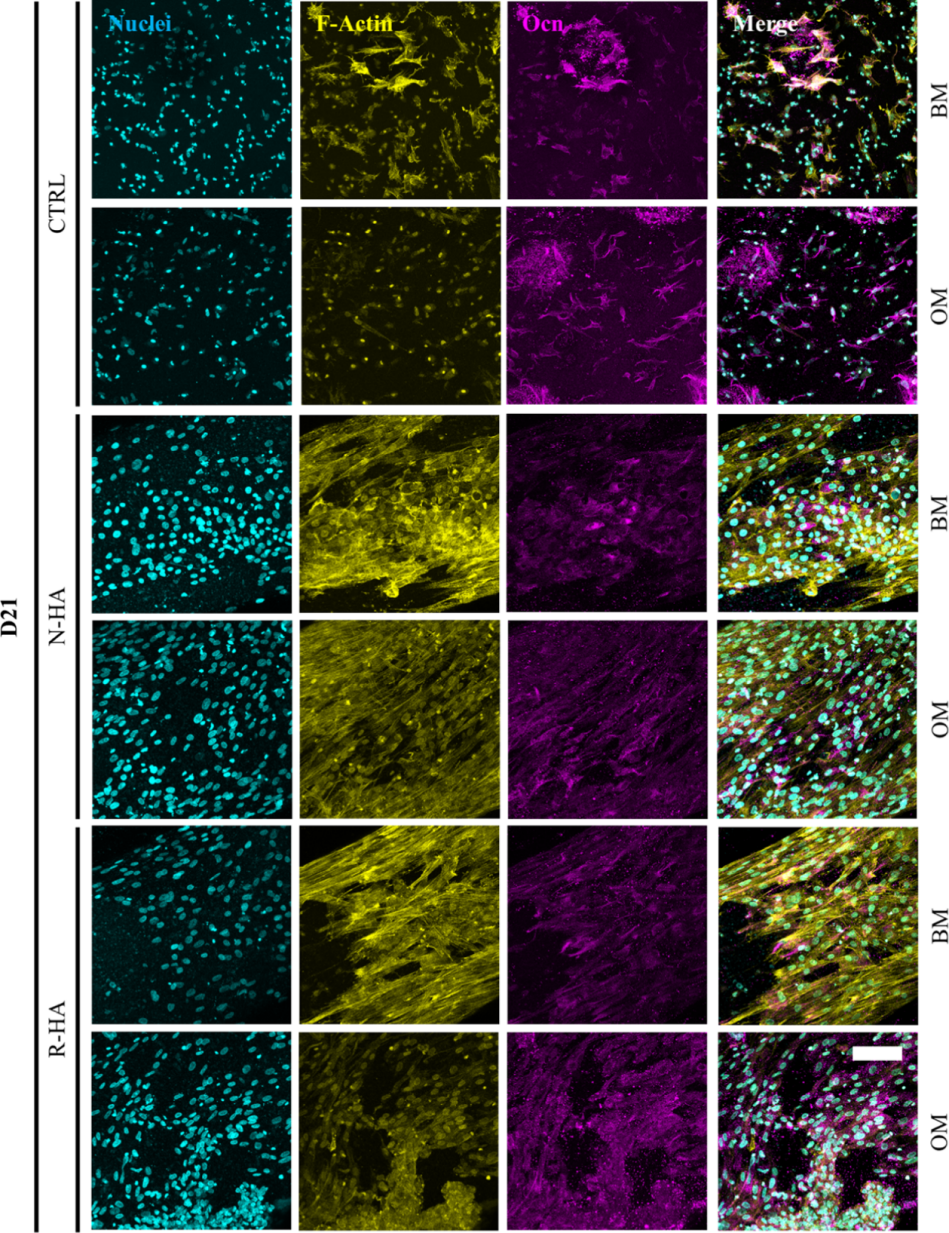
Immunocytochemical evaluation of CTRL, N-HA, and R-HA 3D bioprinted structures at D21 for the late osteogenic marker protein *Ocn*. Staining is presented in split images for nuclei (cyan), F-Actin (yellow), anti-*Ocn* (magenta), and a merge of all channels. Scale bar refers to 100 μm.

Immunofluorescent staining was performed to assess the expression of osteocalcin (*Ocn*) as a late differentiation marker at the D21 level (Figure 5). As expected after the gene expression analysis, *Ocn* was detected in all the samples. However, the production and distribution of *Ocn* varied between the groups. In the CTRL samples, *Ocn* was found in clusters, whereas in nHA groups, *Ocn* was evenly distributed within the 3D bioprinted filaments. As *Ocn* is the most abundant noncollagenous bone protein, secreted in connection to the mineralization phase of osteogenic differentiation, the positive staining may indicate osteoblast presence and osteogenic differentiation. In the CTRL BM samples, the *Ocn* production is limited and localized. However, as the unanticipated *Ocn* expression was confirmed already at an early time point, the protein production could be the result of the stimulation caused by the bioprinting shear stress. The even distribution of *Ocn* in N-HA and R-HA samples is superior to that in CTRL and relates to the presence of nHA, as homogeneously distributed nanoparticles release ions into the surroundings. A similar positive *Ocn* detection under BM conditions was also reported by Kim et al. on D14 with amorphous calcium magnesium phosphate ceramic phase.^22^ The results are most comparable to those of R-HA in the current study. It should be noted that no difference can be noted between the nHA groups.

Although the biological responses differed between N-HA and R-HA at early time points, both ultimately produced uniform *Ocn* expression along the bioprinted filaments under both culture conditions. This indicates that both synthesized biomimetic nHAs show promise as a bioceramic phase in bioinks for bone tissue engineering, as well as potential osteoinductive properties enhanced by stimulation from the bioprinting process. However, more investigations and longer experiments are required to understand and verify the kinetic release of different ions from these nanoparticles and relate that to the cell response.

## 4 Conclusions

In conclusion, two biomimetic nHAs, differing in morphology and physicochemical properties, were synthesized and characterized. These nanoparticles were Mg^2+^- and Mg^2+^/CO_3_^2–^-doped to achieve nature-inspired and biomimetic properties. When incorporated into GelMA-gelatin inks, the shear rate dependency resulted in a decrease in viscosity. Overall, the addition of nHA to the GelMA-gelatin ink improved both the flow and the postprinting stability of the printed structures. The needle-like morphology of N-HA improved printability in terms of pore area accuracy and buildability more effectively than the round particles of R-HA. The ceramic particles did not compromise the cross-linking of GelMA-based samples, and their structural stability was demonstrated throughout the observation period. The viability of bone marrow mesenchymal stromal cells (hBMSCs) was high for all bioinks and demonstrated the most pronounced colonization in N-HA samples. The N-HA increased the metabolic activities (MA) of the cells significantly without osteogenic stimulation, whereas higher MA was shown by the cells exposed to the R-HA and osteogenic medium (OM) at the end of the culture period. For osteogenic differentiation markers, gene expressions of *Runx2* and *ALP* were significantly higher for R-HA on day 21 in OM. However, the expression of *Ocn* was significantly higher for N-HA.

Altogether, the *in vitro* data indicated that the two nHAs interact differently with their environment, underscoring the importance of both the chemistry and morphology of nHA in bioink performance. Optimization of the physicochemical properties of hydroxyapatite nanoparticles seems to be essential for the development of more biomimetic bioinks for advancing bone bioprinting applications.

## Supporting Information Available

Additional experimental details are provided on the cytotoxicity evaluation of nHAs in 2D monoculture system, including live/dead staining and metabolic activity assessment. Additionally, live/dead staining and CCK-8 analysis of bioprinted hBMSCs are presented to assess cell viability and metabolic activity (PDF)
