# Supplementary material for: Effects of Magnesium-Doped Hydroxyapatite Nanoparticles on Bioink Formulation for Bone Tissue Engineering
Source: ACS Appl Bio Mater. 2025 Jan 8;8(1):535–47. doi: 10.1021/acsabm.4c01418 (PMC11752522; doi:10.1021/acsabm.4c01418)
Supplement: Supplementary file 1 — mt4c01418_si_001.pdf [file mt4c01418_si_001.pdf]

Supporting Information

Effects of magnesium-doped hydroxyapatite nanoparticles on bioink formulation for bone tissue engineering

Margherita Montanari[1a], Jannika T. Korkeamäki[2a], Elisabetta Campodoni[1], Samih Mohamed-Ahmed[2], Kamal Mustafa[2*], Monica Sandri[1b*], Ahmad Rashad[2,3b*]

[1]Institute of Science, Technology and Sustainability for Ceramics (ISSMC) – National Research Council (CNR), 48018 Faenza (RA), Italy

[2]Center of Translational Oral Research (TOR), Department of Clinical Dentistry, University of Bergen, 5009, Bergen, Norway

[3]Bioengineering Graduate Program, Aerospace and Mechanical Engineering, University of Notre Dame, Notre Dame, Indiana 46556, United States

[a]Authors contributed equally to this work.

[b]Authors contributed equally to this work.

[*]Corresponding authors:

Kamal Mustafa, kamal.mustafa@uib.no

Monica Sandri, monica.sandri@issmc.cnr.it

Ahmad Rashad, ahmad.elsebahy@uib.no

1. Materials and methods

2D nHA cytotoxicity evaluation

Cytotoxicity of the nHAs was indirectly assessed by leachate treatment on monolayers of hBMSCs. N-HA and R-HA leachates were prepared by magnetic stirring of 1% w/w_{tot} nHA powder in DMEM at 37°C over-night. A sterile filter (0.2 μm) was used to obtain a clear leachate. After filtration, the leachate was completed as basal medium (BM, 10% FBS and 1% P/S) or osteogenic medium (OM; BM supplemented with L-ascorbic acid (173μM), dexamethasone (10nM), and β-glycerophosphate (10mM) (Sigma Aldrich, St. Louis, MO, USA)). HBMSCs cultured with plain (without leachates) BM or OM were used as controls (CTRL). HBMSCs were seeded in 12-well plates with a density of 3 x 10^3 cells/cm^2. HBMSCs were exposed to the leachate 24 h post seeding and cultured for 7 days for cytotoxicity assessment.

Cell viability was assessed by live/dead (L/D, Live/Dead® Viability/Cytotoxicity Kit for mammalian cells, Molecular Probes™, Invitrogen detection technologies) test and the images were taken with a fluorescence microscope (Nikon Eclipse Ti, Tokyo, Japan).

For cell proliferation (n=5), metabolic activity was used as an indicator using Cell Counting Kit-8 (CCK-8, Dojindo Molecular Technologies, Inc., Thermo Fisher Scientific) according to manufacturer's

instructions. The absorbance was read at 450 nm with a microplate reader (Varioskan™ LUX, VLBL00D0, Thermo fisher Scientific).

2. Results

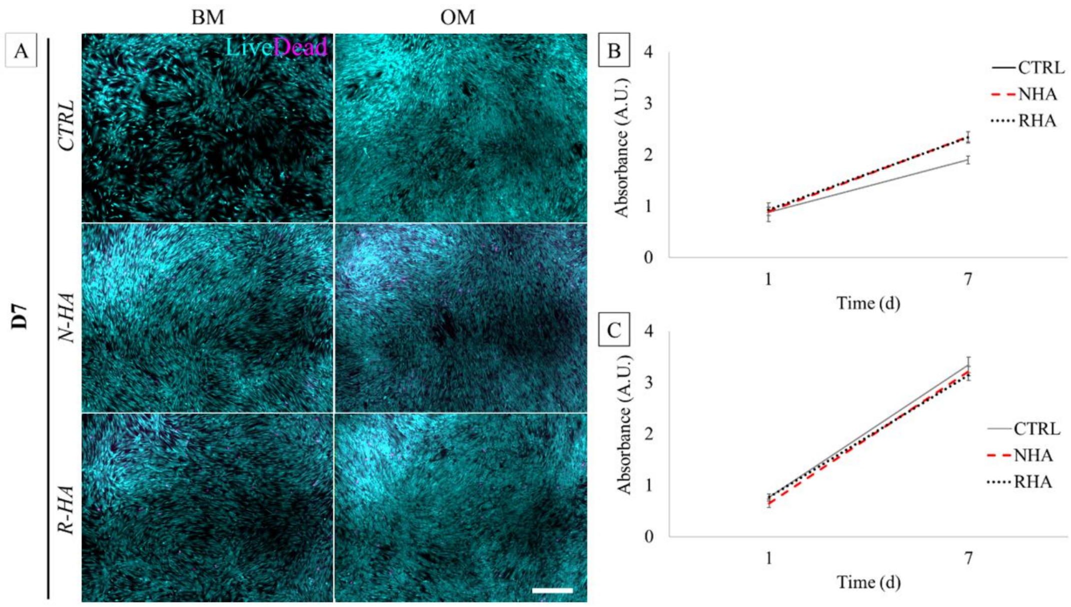

Figure S1 Indirect cytotoxicity evaluation including. A) Cell viability: live (cyan), dead (magenta). Scalebar 500 µm. Metabolic activity of hBMSCs cultured in B) BM and C) OM for 7 days.

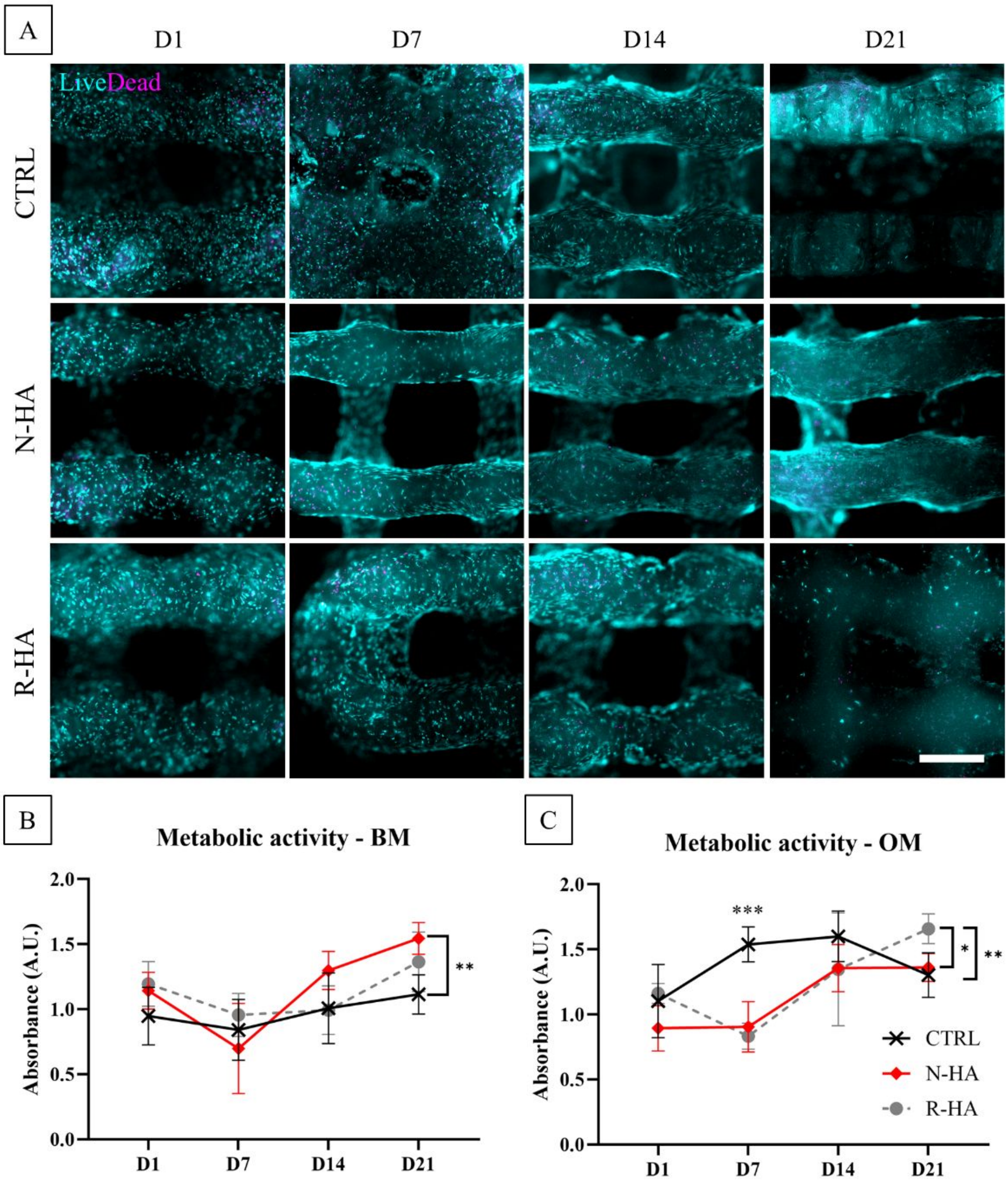

*Figure S2 A) Representative focal planes of cell viability. The hBMSCs were cultured in BM: live (cyan), dead (magenta). Scalebar 500 µm. B) Metabolic activity (n=5) of bioprinted hBMSCs at D1, D7, D14 and D21, cultured in BM and C) OM. The results are presented as absorbance units. Differences between groups: * p<0.05, ** p<0.01, *** p<0.001*
